# Statistical representation models for mutation information within genomic data

**DOI:** 10.1186/s12859-019-2868-4

**Published:** 2019-06-13

**Authors:** N. Özlem ÖZCAN ŞİMŞEK, Arzucan ÖZGÜR, Fikret GÜRGEN

**Affiliations:** Department of Computer Engineering, Boğaziçi University, İstanbul, Turkey

**Keywords:** Information retrieval, Machine learning, tf-idf, tf-rf, BM25, DNA mutations, Gene weighting, Disease classification

## Abstract

**Background:**

As DNA sequencing technologies are improving and getting cheaper, genomic data can be utilized for diagnosis of many diseases such as cancer. Human raw genome data is huge in size for computational systems. Therefore, there is a need for a compact and accurate representation of the valuable information in DNA. The occurrence of complex genetic disorders often results from multiple gene mutations. The effect of each mutation is not equal for the development of a disease. Inspired from the field of information retrieval, we propose using the term frequency (tf) and BM25 term weighting measures with the inverse document frequency (idf) and relevance frequency (rf) measures to weight genes based on their mutations. The underlying assumption is that the more mutations a gene has in patients with a certain disease and the less mutations it has in other patients, the more discriminative that gene is.

**Results:**

We evaluated the proposed representations on the task of cancer type classification. We applied various machine learning techniques using the tf-idf and tf-rf schemes and their BM25 versions. Our results show that the BM25-tf-rf representation leads to improved classification accuracy and f-score values compared to the other representations. The highest accuracy (76.44%) and f-score (76.95%) are achieved with the BM25-tf-rf based data representation.

**Conclusions:**

As a result of our experiments, the BM25-tf-rf scheme and the proposed neural network model is shown to be the best performing classification system for our case study of cancer type classification. This system is further utilized for causal gene analysis. Examples from the most effective genes that are used for decision making are found to be in the literature as target or causal genes.

## Background

Complex diseases with genetic components arise from different combinations of the mutations in DNA. With the help of the decreasing cost of sequencing technologies, large scale sequencing datasets are being curated. Machine learning methods can be helpful in analyzing the huge genome data. However, a suitable representation technique for the sequencing data is still a problem to be solved. In this paper, we propose using statistical methods from the field of information retrieval for the representation of mutation information in DNA. The proposed representation methods are evaluated for the task of cancer type classification.

As stated in the 2017 report of National Center for Health Statistics [1], cancer is the second among top leading causes of death. Cancer is a group of diseases and each cancer type is labeled by the primary area of the body where the cancer cells arise. Each cancer type in general has a different set of causal genes and the disease emerges from the combination of various mutations of these genes [2]. The treatment is planned according to the primary site. Late diagnosis prevents the application of treatments and often results in the loss of the patient. Accordingly, the unknown or wrong analysis of the primary site and late diagnosis are major problems for cancer patients. The use of genomic data for diagnosis might help both to recognize the disease in early stages and to accurately classify the primary site.

Cancer classification has been primarily based on the morphological appearance of the tumor. Medical images such as magnetic resonance images (MRI) [3–5], X-ray and computed tomography (CT) images [6, 7], as well as histopathology images [8, 9] have been utilized for cancer diagnosis and classification. Medical images provide valuable information, especially about tumors, but they represent a restricted area. Therefore, there is a need for a more comprehensive data source.

Another commonly used data type for cancer classification is gene expression data. While a number of studies utilizing gene expression data have addressed the classification of cancer types [10–14], this type of data is highly sensitive to the microarray experiment setup and in general suffers from low accuracy and robustness [15]. In addition, due to the high dimensionality of gene expression data, gene selection methods are commonly applied prior to classification [16, 17]. The feature selection step may eliminate genes that in general have minor effects on disease generation while still being significant for the diagnosis of particular cancer types for some patients.

The biotechnology improvements and the automation of sequencing systems have increased the speed and lowered the cost of human DNA sequencing, which enabled the usage of this data type for disease diagnosis. The variants or mutations in the DNA of an individual can be identified by comparing the DNA sequence of the individual to the DNA sequence of a reference genome maintained by The Genome Reference Consortium [18] and stored in a variant call format (VCF) file [19]. In recent studies, the binary representations of mutation data obtained from sources of manually curated somatic mutation profiles have been utilized for cancer classification [20, 21]. However, binary representation is a limited way of describing the data. It highlights the genes with mutations, but treats them as equal. The distinction of common, rare and disease causing mutations is not expressed with the binary representation. Therefore, methods such as C-score from the Combined Annotation Dependent Depletion (CADD) framework [22, 23] have been developed for weighting gene mutations. Recently, the sum of C-scores of gene mutations has been successfully used to cluster breast cancer patients and predict the stage of the disease [24].

In this paper, we propose adapting and using term weighting techniques (tf-idf, tf-rf and BM25) from the information retrieval field for weighting genes based on mutation information. As far as we know, these techniques have not been used on variant data before. The proposed gene weighting techniques are evaluated for the task of cancer type classification. Our results demonstrate that the best performing information retrieval based model (BM25-tf-rf) outperforms the C-score based approach. When the best performing classification model is analyzed, the most effective genes in the classification of certain cancer types are found to have been also proposed as causal or target genes in the previously published studies. These literature findings support the effectiveness of our representation models.

Our work brings the following contributions:

1. Term weighting methods from the field of information retrieval have been proposed for the representation of mutation information within genomic data.
2. A comparison of these data representation schemes for the task of cancer type classification has been performed using a wide range of machine learning methods.
3. The best performing representation and classification model are utilized for causal gene analysis.

## Methods

**Fig. 1:**
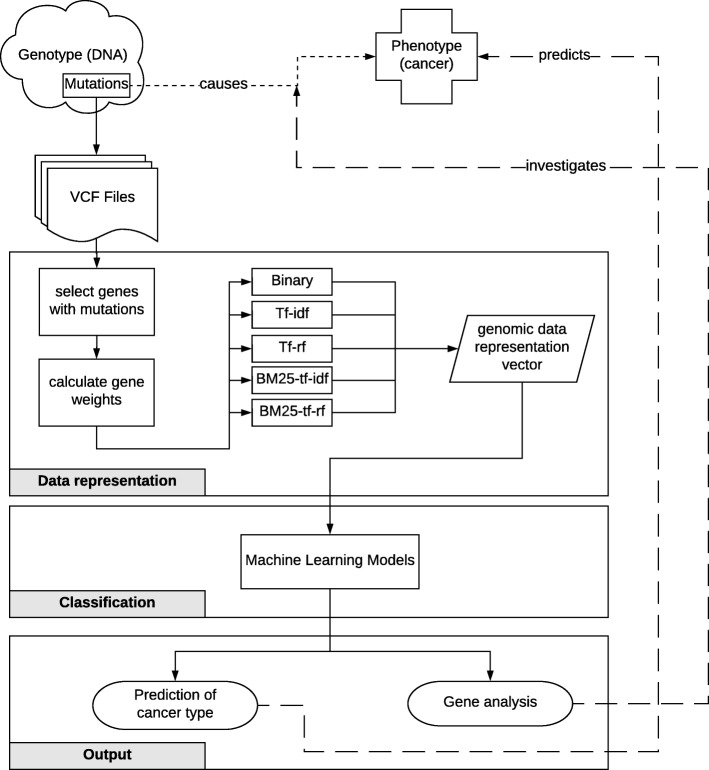
Overview of the proposed system

In this section, we describe the proposed data representation models as well as their utilization with machine learning algorithms for cancer type classification. An overview of the developed system is shown in Fig. 1. The phenotypes occur as a result of DNA mutations. In our system, we take VCF files, which hold the DNA mutation information, as input. These gene mutations are weighted by using the proposed representation models. The genomic data representation vector is then processed with a wide range of machine learning algorithms for the task of cancer type classification. So, the VCF data constitute the set of observations and the genes, which are weighted based on mutation information, are the features in our classification model for learning the given cancer classes. The first output of the system is the prediction of the cancer type. The second output is the list of most effective genes in the classification process, which is obtained by analyzing the most accurate representation and classification model.

### Dataset

The Cancer Facts and Figures 2017 annual report [25] states the leading sites of cancer. According to this list and the common cancer types from The National Cancer Institute [2], we created a dataset of ten cancer types, which are observed frequently and account for above half of the estimated cancer caused deaths. The list of selected cancer types and the sample counts for each cancer type are provided in Table 1. We downloaded a total of 7028 VCF files for ten cancer types from The Cancer Genome Atlas (TCGA) [26]. ANNOVAR [27] is used for gene-based annotation of the VCF files. From the annotated files, we selected the exonic and intronic mutations as they include specific gene labels. This selection of mutation types resulted in 16,383 distinct genes. As a result, our dataset, named as BOUN10CANCER, has 7028 samples with mutation information of 16,383 distinct genes and a class label for each sample representing the cancer type.

**Table 1:** The list of cancer types and sample counts in the BOUN10CANCER dataset

Cancer type	Sample count
Lung	1232
Breast	1080
Brain	1028
Kidney	734
Colorectal	656
Thyroid	504
Prostate	503
Skin	472
Stomach	441
Liver	378

### Data representation models

#### Binary mutation model

In the binary model, a gene is represented with 1 if there is a mutation in that gene, and it is represented with 0 otherwise. Hence, the resulting data set has the gene labels as features and binary values for each feature. The binary value for each gene feature is extracted from the annotated VCF file. If the gene label exists in the annotated file, the value is 1, and it is 0 otherwise. If there are more than 1 mutation for a gene, the feature value for that gene is still set to 1.

This representation model is applied by using two different gene lists. The first model is constructed with the known causal genes for the selected cancer types. The causal genes are obtained from OMIM [28] by using the MSC tool [29] of HGNC [30]. We extracted 434 causal genes for the cancer types in the BOUN10CANCER dataset. The second model is constructed by using all mutated genes in the annotated VCF files. We extracted 16,383 mutated genes for the cancer types in the BOUN10CANCER dataset.

#### C-score based mutation model

The CADD framework [22, 23] is a Support Vector Machine (SVM) based framework which calculates C-scores for variants. The C-score integrates diverse annotations and creates a single score for a variant. In the C-score based mutation model, the sum of C-scores for all mutations in a gene is used as the feature value for that gene. For example, if a gene has two mutations in a sample, the feature value for that gene in that sample is the sum of the C-scores of these two mutations. The sum of C-scores approach was also used in [24] for breast cancer patient classification. We evaluate this approach for cancer type classification and compare it with the proposed information retrieval based mutation models described in the following subsections.

#### Tf-idf based mutation model

Complex diseases are in general developed from the combination of various mutations in the genes. Each mutation may influence the evolution of the disease at different levels. In order to express these differences, we proposed to utilize the tf-idf (term frequency-inverse document frequency) weighting method. Tf-idf is a term weight calculation technique used commonly in the information retrieval and text mining research areas. In [31], tf-idf is defined as a statistical measure, which is used to evaluate how important a word is to a document in a collection by checking the distribution and frequency of the word’s occurrences.

In our context, the tf-idf value measures how important a gene mutation is to a sample in a collection of samples. Mutations in genes that are found in most samples have low tf-idf values, whereas genes with rare mutations are granted higher weights. With this strategy, we aim to increase the impact of the existence of rare mutations and suppress the effects of common mutations in the classification task, since common mutations may not be a sign of a disease.

In this model, instead of binary values, the calculated tf-idf weights of the genes are used as feature values. The main equation of tf-idf is presented in Eq. 1. Tf-idf value for a gene *g* and sample *s* is the multiplication of tf, that is term frequency, and idf, that is inverse document frequency, values. The tf value for a gene *g* and sample *s* is taken as the count of mutations of gene *g* in sample *s*. The higher the number of mutations for a gene in a sample, the more tf weight is assigned to this gene. The df value, that is document frequency, for a gene *g* is taken as the count of samples in the collection that contain mutations of gene *g*. For a sample collection of size *N*, idf of gene *g* is calculated as shown in Eq. 2. Intuitively, the more samples in the collection have mutations in gene *g*, the less discriminating power this gene will have as a feature in cancer type classification. So it is assigned a lower idf score.

1 $$ {tf\text{-}idf}_{g,s} ={tf}_{g,s}*{idf}_{g}   $$

2 $$ {idf}_{g} = \log \left(N / {df}_{g}  \right)  $$

#### Tf-rf based mutation model

A mutation can be rare in the collection, however, it may be effective for samples with particular cancer types. In order to account for the class information, tf-rf (term frequency-relevance frequency) based data representation is adapted. Similarly to tf-idf, tf-rf is also used in information retrieval and text mining. Unlike tf-idf, tf-rf is a supervised statistical measure proposed in [32]. It is used to evaluate how important a word is to a class of documents in a collection. In tf-rf, a word may have different weight values for different classes.

In our context, the tf-rf value measures how important a gene mutation is to a sample by using the information of its class label. If the particular gene mutation is encountered more in one class compared to the other classes, the corresponding rf and tf-rf values are higher than for the other classes. As shown in Eq. 3, the tf-rf value for a gene *g* and sample *s* is the multiplication of tf, that is term frequency, and rf, that is relevance frequency, values. The tf value is computed in the same way as in tf-idf. The rf value of gene *g* and class *c* is calculated as in Eq. 4, where *a* is the number of samples in class *c* which contain mutation in gene *g*, and *b* is the number of samples in other classes which contain mutation in gene *g*.

3 $$ {tf\text{-}rf}_{g,s} ={tf}_{g,s}*{rf}_{g,c}   $$

4 $$ {rf}_{g,c} =\log (2 + a / \max (1,b))   $$

#### BM25-tf-idf based mutation model

BM25, often called Okapi, is a ranking function used by search engines to rank matching documents according to their relevance to a given search query [33]. For our task of weighting genes based on mutation information, the term frequency definition in BM25 is used instead of the classic term frequency in tf-idf. As shown in Eq. 5, BM25-tf-idf value for a gene *g* and sample *s* is the multiplication of BM25-tf, that is BM25 definition of term frequency, and *idf*, that is inverse document frequency, values. BM25-tf value for a gene *g* and sample *s* is calculated as in Eq. 6. In this equation, *L*_*s*_ and *L*_*ave*_ are the length of sample *s* and the average sample length for the whole collection, respectively. We model the samples with the same features. Therefore, in our representation model, *L*_*s*_ is equal to *L*_*ave*_. When we use this equality, Eq. 6 is simplified to Eq. 7. *k* is used as a smoothing parameter for *tf*. The *idf* definition is the same as in tf-idf.

5 $$  {BM25\text{-}tf\text{-}idf}_{g,s} = {BM25\text{-}tf}_{g,s} * {idf}_{g}  $$

6 $$ {{}\begin{aligned} {BM25\text{-}tf}_{g,s} &= \left((k+1)*{tf}_{g,s}\right) / \left(k*\left((1-b)\right. \right.\\&\quad \left. \left. +b*(L_{s}/L_{ave})\right)+{tf}_{g,s}\right) \end{aligned}}  $$

7 $$  {BM25\text{-}tf}_{g,s} = ((k+1)*{tf}_{g,s}) / (k+{tf}_{g,s})  $$

#### BM25-tf-rf based mutation model

For BM25-tf-rf, the term frequency definition in BM25 is used instead of the classic term frequency in tf-rf. As shown in Eq. 8, the BM25-tf-rf value for a gene *g* and sample *s* is the multiplication of BM25-tf, that is BM25 definition of term frequency, and *rf*, that is relevance frequency, values. The BM25-tf value is computed in the same way as in BM25-tf-idf. The *rf* definition is the same as in tf-rf.

8 $$  {BM25\text{-}tf\text{-}rf}_{g,s} = {BM25\text{-}tf}_{g,s} * {rf}_{g,c}  $$

**Fig. 2:**
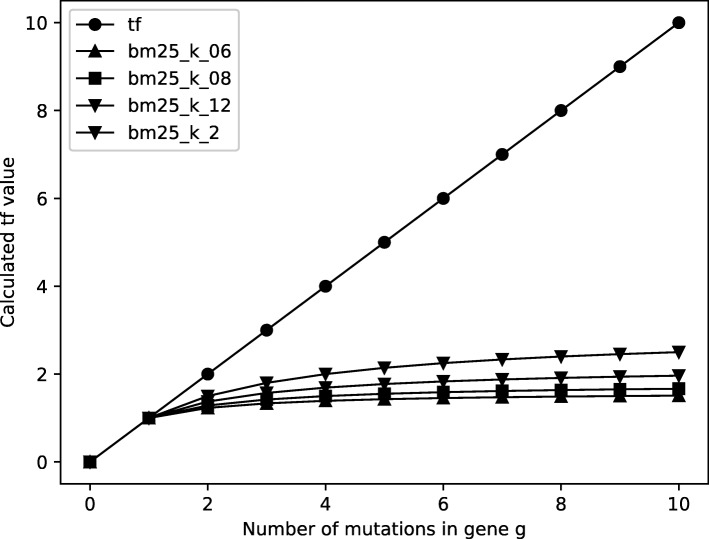
The effect of the smoothing parameter k in the BM25 calculations for term frequency

The effect of the smoothing parameter k is illustrated in Fig. 2. In this chart, the *tf* and *BM*25-*tf* values for different values of k are shown when the number of gene mutations changes in the range from 1 to 10. The figure demonstrates that, the *tf* values, which are represented by empty circles, keep increasing as the number of mutations increases. Even a point mutation may be significant for the occurrence of a certain disease. Therefore, a gene with *n* mutations is not necessarily *n* times more important than a gene with 1 mutation for disease detection. As shown in Fig. 2 the smoothing parameter *k* in *BM*25-*tf* dampens the effect of high *tf* values.

### Implementation and experiment design

#### Machine learning models

A wide range of machine learning algorithms are applied to investigate the effects of the proposed mutation based DNA representation models in the task of disease classification. Naive Bayes (NB), K-Nearest Neighbor (KNN), Support Vector Machines (SVM), Logistic Regression (LR), One-Layer-Perceptron (Perceptron) and Feed-Forward Multilayer Neural Network (NN) are run on the prepared datasets.

For the Feed Forward NN, the model is composed of two or more fully connected layers. Except the last layer, the number of nodes is halved at each layer. If the first layer has N units, then the second layer has N/2 units. With this strategy, each layer represents the information from the previous layer with less units. After each fully connected layer, a dropout is applied. As there are 10 classes, the last layer has 10 nodes with softmax activation function. Categorical cross-entropy is employed as the loss function. The number of epochs is 50 and the batch size is 50.

All experiments are implemented with Python. For the traditional machine learning algorithms, the scikit-learn library [34] is used. The feed forward network model is implemented with Keras [35] on Tensorflow [36] backend.

#### Evaluation strategy

The input datasets are first divided into 80% training and 20% test sets. Parameter tuning is accomplished using 10-fold cross-validation on the training set. Testing is also accomplished in 10-folds. In each fold, the model with the best parameters is trained with one of the training sets from the initial cross-validation experiment, which was performed over the 80% of the data, and testing is performed over the test set (the initially separated 20% of the data). By this strategy, the instances in the independent test set are not included in any step of either parameter tuning or training, and the effects of minor changes of the training data are also indicated. The reported results are the micro-averaged scores and standard deviations on the independent test set. Accuracy, f-score, precision, recall, false positive rate (FPR) and area under the receiver operating curve (roc-auc) are used as the performance measures.

#### Parameter tuning for the representation models

For BM25-tf, a range of k values between 0.6 and 2 are used in the parameter tuning phase. The BM25-tf-rf representation model and the Feed Forward NN are used in the parameter tuning setup. The classification results for different values of k are presented in Table 2 and the best performing row is shown in bold. k =0.8 leads to the best accuracy and f-score values. Therefore, this value is used in our experiments for the BM25-tf calculations.

**Table 2:** Parameter tuning results for the parameter k in the BM25-tf formula

k	Accuracy	F-Score	Precision	Recall
0.6	75.20 ± 1.21	75.89 ± 1.14	76.59 ± 1.11	75.20 ± 1.21
**0.8**	**75.38** ± **1.02**	**76.18** ± **1.03**	**76.99** ± **1.10**	**75.38** ± **1.02**
1.0	75.24 ± 1.60	75.87 ± 1.55	76.51 ± 1.53	75.24 ± 1.60
1.2	74.60 ± 1.60	75.32 ± 1.49	76.07 ± 1.39	74.60 ± 1.60
1.4	74.24 ± 1.00	74.88 ± 0.76	75.54 ± 0.65	74.24 ± 1.00
1.6	74.43 ± 1.39	75.35 ± 1.28	76.30 ± 1.42	74.43 ± 1.39
1.8	74.21 ± 1.46	74.89 ± 1.40	75.58 ± 1.38	74.21 ± 1.46
2.0	74.73 ± 1.32	75.53 ± 1.21	76.36 ± 1.16	74.73 ± 1.32

The row with the highest scores is shown in bold.

#### Parameter tuning for the classification models

A parameter tuning phase is applied for each machine learning algorithm and data representation model. The best parameter set is used in the test phase. The default values are used for the parameters that are not tuned. NB and LR are applied with the default parameters. The range or list of values used in parameter tuning for the other machine learning algorithms are presented in Table 3. The best parameters for the classification models are presented in Table 4.

**Table 3:** The range (or list) of parameters used in the parameter tuning phase for the classification models

Algorithm	Parameter	Range or Values
KNN	k	[2,150]
SVM	Kernel	linear, polynomial, rbf
	Polynomial degree	[2,5]
	Gamma	[10^−4^,10^−1^]
	Cost	[10^1^,10^4^]
Perceptron	Optimization function	Adam, SDG
	Activation function	ReLU, tanh
	Hidden layer size	[10,100]
	The maximum number of iterations	[100,300]
Feed Forward NN	Optimization function	Adam, SDG
	Activation function	ReLU, tanh
	The number of layers	[2,6]
	Dropout rate	[0.25,0.5]
	The number of nodes in the first layer	[1024,8192]

**Table 4:** The best parameters found as a result of the parameter tuning phase for the classification models

Algorithm	Parameter	Value	Data Rep.
KNN	k	50	Binary
		10	c-score, tf-idf, tf-rf,
			bm25-tf-idf, bm25-tf-rf
SVM-poly	Polynomial degree	3	binary, tf-idf, bm25-tf-idf
		2	c-score, tf-rf, bm25-tf-rf
SVM-rbf	Gamma	10^−4^	All
	Cost	10^3^	All
SVM-linear	Gamma	10^−4^	All
	Cost	10^2^	All
Perceptron	Optimization function	SGD	binary
		Adam	c-score, tf-idf, tf-rf,
			bm25-tf-idf, bm25-tf-rf
	Activation function	tanh	binary
		ReLU	c-score, tf-idf, tf-rf,
			bm25-tf-idf, bm25-tf-rf
	Hidden layer size	100	All
	The maximum number of iterations	200	binary
		300	c-score, tf-idf, tf-rf,
			bm25-tf-idf, bm25-tf-rf
Feed Forward NN	Optimization function	Adam	All
	Activation function	ReLU	All
	The number of layers	4	All
	Dropout rate	0.25	All
	The number of nodes in the first layer	8192	All

## Results

### Selection of gene sets

The success of the mutation based data representation schemes also depends on the selected gene list. The initial experiments are applied on the binary representation to observe the classification performances over the causal and full gene sets. LR, SVM with linear kernel and Perceptron are selected as pilot algorithms. As shown in Table 5, the accuracy values are between 33% and 37% for the binary causal gene dataset and between 66% and 69% for the binary full gene dataset. It is observed that, the addition of extra gene information nearly doubles the classification accuracy. The mutation data of the additional genes increases the accuracy. The dramatic increase can be observed in f-score results too. This result can be interpreted as being an indication of the existence of new (currently unknown) causal genes. As the accuracy of the classification models are enhanced with additional genes, the rest of the experiments are applied on the full gene datasets.

**Table 5:** Machine learning experiment test results on the gene sets with the binary representation model

Gene Set	Algorithm	Data Rep.	Accuracy	F-Score	Precision	Recall	Roc-Auc	FPR
causal	LR	binary	36.81 ± 0.45	36.36 ± 0.50	35.93 ± 0.52	36.81 ± 0.45	0.63 ± 0.03	9.03 ± 0.10
causal	SVM-linear	binary	33.53 ± 0.32	32.70 ± 0.99	31.92 ± 1.13	33.53 ± 0.32	0.62 ± 0.05	9.38 ± 011
causal	Perceptron	binary	36.74 ± 0.56	36.62 ± 0.83	36.52 ± 2.56	36.74 ± 0.56	0.63 ± 0.06	10.01 ± 0.10
all	LR	binary	67.19 ± 0.41	68.01 ± 0.01	68.01 ± 0.00	67.01 ± 0.01	0.78 ± 0.01	3.85 ± 0.07
all	SVM-linear	binary	68.46 ± 0.67	68.01 ± 0.01	69.01 ± 0.01	68.01 ± 0.01	0.78 ± 0.01	4.07 ± 0.09
all	Perceptron	binary	68.50 ± 0.48	69.01 ± 0.01	70.01 ± 0.01	68.01 ± 0.01	0.78 ± 0.03	4.07 ± 0.09

### Comparison of the data representation models with machine learning experiments

Machine learning algorithms are applied in order to explore the effects of the proposed data representations. Table 6 lists the results of the machine learning experiments. The table is designed to compare the data representation models for each algorithm. The row with the best result is shown in italic for each algorithm and the overall best performance is made bold.

**Table 6:** Machine learning experiment test results on the data representation models of the full gene BOUN10CANCER dataset

Algorithm	Data Rep.	Accuracy	F-Score	Precision	Recall	Roc-Auc	FPR
NB	binary	33.84 ± 0.83	35.25 ± 0.95	37.04 ± 1.34	33.84 ± 0.83	0.62 ± 0.02	8.38 ± 0.11
	c-score	31.10 ± 0.86	32.72 ± 0.74	34.53 ± 1.43	31.10 ± 0.86	0.59 ± 0.01	8.61 ± 0.08
	tf-idf	33.34 ± 0.48	35.04 ± 0.60	37.03 ± 1.03	33.34 ± 0.48	0.62 ± 0.02	7.99 ± 0.07
	tf-rf	*38.14 ± 0.57*	*38.97 ± 0.87*	*40.08 ± 1.27*	*38.14 ± 0.57*	*0.65 ± 0.01*	*7.99 ± 0.10*
	bm25-tf-idf	32.50 ± 0.96	34.19 ± 0.87	36.08 ± 1.35	32.50 ± 0.96	0.60 ± 0.01	8.48 ± 0.10
	bm25-tf-rf	37.94 ± 0.63	38.99 ± 0.60	40.12 ± 1.24	37.94 ± 0.63	0.62 ± 0.01	7.91 ± 0.10
KNN	binary	11.54 ± 0.85	16.87 ± 0.66	31.46 ± 2.54	11.54 ± 0.85	0.50 ± 0.04	7.41 ± 0.04
	c-score	15.87 ± 0.63	22.60 ± 0.44	39.27 ± 4.21	15.87 ± 0.63	0.53 ± 0.01	7.96 ± 0.07
	tf-idf	*34.96 ± 0.66*	*37.35 ± 0.95*	*38.92 ± 0.69*	*34.96 ± 0.66*	*0.62 ± 0.03*	*8.10 ± 0.04*
	tf-rf	19.29 ± 0.44	22.23 ± 0.61	40.29 ± 0.82	19.29 ± 0.44	0.55 ± 0.02	7.57 ± 0.07
	bm25-tf-idf	12.72 ± 1.23	20.05 ± 0.58	47.32 ± 5.85	12.72 ± 1.23	0.51 ± 0.01	8.17 ± 0.37
	bm25-tf-rf	11.91 ± 1.13	19.21 ± 0.50	49.74 ± 1.58	11.91 ± 1.13	0.51 ± 0.01	7.88 ± 0.17
SVM-poly	binary	17.50 ± 0.00	5.21 ± 0.00	3.06 ± 0.00	17.50 ± 0.00	0.53 ± 0.00	16.34 ± 0.00
	c-score	*56.14 ± 0.44*	*58.90 ± 0.39*	*61.96 ± 0.46*	*56.14 ± 0.44*	*0.73 ± 0.01*	*5.33 ± 0.06*
	tf-idf	17.50 ± 0.00	5.21 ± 0.00	3.06 ± 0.00	17.50 ± 0.00	0.53 ± 0.00	16.35 ± 0.00
	tf-rf	55.51 ± 0.55	56.52 ± 0.65	61.40 ± 0.53	55.51 ± 0.55	0.71 ± 0.03	5.16 ± 0.05
	bm25-tf-idf	36.36 ± 0.66	42.64 ± 0.75	51.56 ± 0.89	36.36 ± 0.66	0.62 ± 0.01	7.93 ± 0.08
	bm25-tf-rf	53.41 ± 0.27	51.46 ± 0.27	63.95 ± 0.65	53.41 ± 0.27	0.66 ± 0.01	7.38 ± 0.04
SVM-rbf	binary	66.71 ± 0.36	67.01 ± 0.00	68.01 ± 0.00	67.01 ± 0.01	0.78 ± 0.01	4.04 ± 0.09
	c-score	57.35 ± 0.30	61.31 ± 0.28	65.86 ± 1.10	57.35 ± 0.30	0.72 ± 0.01	7.09 ± 0.05
	tf-idf	50.92 ± 0.19	44.26 ± 0.20	51.64 ± 0.19	50.92 ± 0.19	0.69 ± 0.02	8.30 ± 0.03
	tf-rf	69.53 ± 0.71	69.82 ± 0.72	70.75 ± 0.71	69.53 ± 0.71	0.78 ± 0.03	3.64 ± 0.09
	bm25-tf-idf	66.17 ± 0.56	66.61 ± 0.60	67.20 ± 0.62	66.17 ± 0.56	0.78 ± 0.01	4.40 ± 0.07
	bm25-tf-rf	*73.77 ± 0.46*	*74.00 ± 0.46*	*74.96 ± 0.40*	*73.77 ± 0.46*	*0.83 ± 0.01*	*3.20 ± 0.07*
SVM-linear	binary	68.46 ± 0.67	68.01 ± 0.01	69.01 ± 0.01	68.01 ± 0.01	0.78 ± 0.01	4.07 ± 0.09
	c-score	71.91 ± 0.44	72.46 ± 0.45	73.02 ± 0.44	71.91 ± 0.44	0.82 ± 0.01	3.50 ± 0.09
	tf-idf	69.54 ± 0.66	69.01 ± 0.01	70.01 ± 0.01	69.01 ± 0.01	0.78 ± 0.01	3.94 ± 0.06
	tf-rf	68.80 ± 0.62	68.01 ± 0.01	69.51 ± 0.01	69.01 ± 0.01	0.78 ± 0.01	3.74 ± 0.09
	bm25-tf-idf	66.26 ± 0.58	66.35 ± 0.60	67.94 ± 0.66	66.26 ± 0.58	0.78 ± 0.01	4.31 ± 0.07
	bm25-tf-rf	*73.44 ± 0.43*	*73.66 ± 0.45*	*74.63 ± 0.41*	*73.44 ± 0.43*	*0.83 ± 0.01*	*3.24 ± 0.07*
LR	binary	67.19 ± 0.41	68.01 ± 0.01	68.01 ± 0.00	67.01 ± 0.01	0.78 ± 0.01	3.85 ± 0.07
	c-score	73.50 ± 0.64	73.89 ± 0.92	74.29 ± 0.66	73.50 ± 0.64	0.83 ± 0.01	3.40 ± 0.08
	tf-idf	63.17 ± 0.30	60.01 ± 0.00	66.01 ± 0.01	63.01 ± 0.00	0.74 ± 0.01	5.68 ± 0.04
	tf-rf	71.51 ± 0.46	72.01 ± 0.01	73.01 ± 0.01	71.01 ± 0.01	0.81 ± 0.01	3.24 ± 0.07
	bm25-tf-idf	67.80 ± 0.45	68.20 ± 0.47	68.61 ± 0.53	67.80 ± 0.45	0.79 ± 0.01	4.09 ± 0.06
	bm25-tf-rf	*74.99 ± 0.41*	*75.19 ± 0.38*	*75.96 ± 0.37*	*74.99 ± 0.41*	*0.83 ± 0.01*	*3.03 ± 0.06*
Perceptron	binary	68.50 ± 0.48	69.01 ± 0.01	70.01 ± 0.01	68.01 ± 0.01	0.78 ± 0.03	4.07 ± 0.09
	c-score	71.64 ± 1.54	71.76 ± 1.87	71.89 ± 1.38	71.64 ± 1.54	0.81 ± 0.01	3.67 ± 0.24
	tf-idf	70.23 ± 0.40	70.01 ± 0.00	70.01 ± 0.01	70.01 ± 0.01	0.79 ± 0.01	3.83 ± 0.05
	tf-rf	72.07 ± 1.86	72.01 ± 0.02	74.01 ± 0.01	72.01 ± 0.02	0.82 ± 0.02	3.29 ± 0.12
	bm25-tf-idf	65.52 ± 0.52	65.97 ± 0.52	66.44 ± 0.56	65.52 ± 0.52	0.78 ± 0.01	4.48 ± 0.08
	bm25-tf-rf	*74.15 ± 0.51*	*74.48 ± 0.56*	*75.46 ± 0.56*	*74.15 ± 0.51*	*0.83 ± 0.01*	*3.07 ± 0.10*
Feed-Forward NN	binary	69.00 ± 0.76	69.52 ± 0.70	71.00 ± 0.52	69.00 ± 0.81	0.79 ± 0.02	3.65 ± 0.17
	c-score	73.74 ± 0.88	74.07 ± 0.73	74.41 ± 0.67	73.74 ± 0.88	0.84 ± 0.02	3.27 ± 0.24
	tf-idf	62.91 ± 0.79	63.32 ± 0.70	65.04 ± 0.52	62.91 ± 0.83	0.73 ± 0.02	4.00 ± 0.10
	tf-rf	74.13 ± 1.33	74.17 ± 1.47	75.43 ± 1.07	74.13 ± 1.40	0.85 ± 0.02	3.07 ± 0.24
	bm25-tf-idf	68.18 ± 1.83	68.79 ± 1.28	69.42 ± 0.76	68.18 ± 1.83	0.82 ± 0.02	4.07 ± 0.54
	bm25-tf-rf	**76.44** ± **0.66**	**76.95** ± **0.68**	**77.48** ± **0.78**	**76.44** ± **0.66**	**0.86** ± **0.02**	**2.75** ± **0.13**

The row with the best accuracy and f-score is shown in italic for each algorithm. The overall best performance is made bold

The accuracy scores of NB, KNN and SVM with polynomial kernel are below 57% and the f-score results are below 59%. The remaining 5 algorithms obtain accuracy and f-score levels above 60% (except SVM-rbf with tf-idf). We will focus on these better performing algorithms. The BM25-tf-rf representation scheme leads to the best accuracy and f-score results for all of the 5 algorithms. In addition, the BM25-tf-rf data representation results in nearly 2 to 4 percent accuracy and f-score improvement and 0.01 to 0.05 roc-auc improvement compared to the second best representation for all of the 5 successful algorithms. When we consider the FPR results, BM25-tf-rf leads to lowest values in all of the 5 successful algorithms.

For the Feed-Forward NN f-score results with binary and BM25-tf-rf representations, the paired t-test produces a *p*-value < 2.5*e*−10, and with the tf-rf and BM25-tf-rf representations, the paired t-test produces a *p*-value < 1.1*e*−05. These results show that the additional statistical information hidden in the BM25-tf-rf representation provides significant gain compared to the other representation models. When we compare the Feed-Forward NN f-score values for the C-score and BM25-tf-rf models, the paired t-test produces a *p*-value < 3.2*e*−06. This significant difference states that, although the BM25-tf-rf scheme doesn’t utilize the various properties of mutations that are expressed in C-score, it is more successful for the differentiation of cancer types with its class-based statistical approach for the mutations. We can conclude that the BM25-tf-rf scheme is a suitable representation tool for the mutation information in VCF files for the cancer type classification task.

The most accurate algorithm (76.44% accuracy and 76.95% f-score) is the Feed-Forward NN with the BM25-tf-rf representation scheme, despite the extra network cost. The precision and recall results for the NN on BM25-tf-rf representation are similar with the accuracy value. The roc-auc result is also the highest compared to the other results in Table 6.

For the LR and NN f-score results with the BM25-tf-rf representation scheme, the paired t-test produces a *p*-value < 2.35*e*−05. For the Perceptron and NN f-score results with BM25-tf-rf, the paired t-test produces a *p*-value < 1.7*e*−06. Thus, the multilayer feed forward neural network model is found to be significantly more accurate than the single layer perceptron and LR with BM25-tf-rf.

### Class-based comparison of experiment results

BM25-tf-rf based representation leads to improved performance results compared to the other representations with almost all machine learning algorithms in our experiments. In addition, the multilayer feed forward neural network model achieves better cancer type classification performance compared to the other machine learning algorithms in our experiments for all data representations except tf-idf. Therefore, we used the results for the NN algorithm with the BM25-tf-rf representation model for further discussions on class-level performance.

Table 7 lists the class-based performance metrics. The cancer types are presented in the order of descending sample count. From this list, it is observed that the classification performance differs for each class. The results show that the success level does not entirely depend on the number of samples in the dataset, as there are fewer samples for Skin cancer than Thyroid cancer, but the f-score for Skin cancer is higher. The proposed model doesn’t perform well for cancer types such as Prostate and Thyroid. Other cancer types such as Lung, Breast, Colorectal, and Skin are classified with better f-scores. This suggests that there may be more distinctive and class specific mutations in these cancer types, which the BM25-tf-tr scheme can model successfully.

**Table 7:** Class based experiment test results with NN on full gene BM25-tf-rf dataset

Cancer Type	F-Score	Precision	Recall	FPR
Lung	85.47 ± 1.20	88.03 ± 2.00	83.16 ± 1.94	2.42 ± 0.48
Breast	95.92 ± 1.81	94.23 ± 2.41	97.69 ± 1.44	1.09 ± 0.47
Brain	69.80 ± 1.23	64.19 ± 3.47	77.13 ± 1.46	2.61 ± 2.32
Kidney	68.51 ± 1.14	73.59 ± 3.48	64.23 ± 2.22	2.72 ± 0.48
Colorectal	88.89 ± 1.92	88.21 ± 3.45	89.93 ± 2.89	1.28 ± 0.66
Thyroid	51.40 ± 3.35	47.86 ± 4.29	56.54 ± 4.43	14.79 ± 1.25
Prostate	39.80 ± 2.28	37.97 ± 4.03	42.32 ± 2.41	15.36 ± 1.03
Skin	89.56 ± 1.21	95.66 ± 3.49	84.38 ± 2.09	1.29 ± 0.26
Stomach	60.30 ± 2.33	74.45 ± 4.41	51.86 ± 4.50	10.31 ± 0.84
Liver	71.51 ± 2.20	83.98 ± 3.67	63.28 ± 4.00	7.75 ± 0.47

### Location-based comparison of gene mutations

In the previous sections all exonic and intronic mutations in the dataset have been utilized to compare the data representation and classification models. The results have shown that the BM25-tf-rf is the best performing representation model and the multilayer feed forward neural network is the best performing classification model in our experiments. By using these best models, a new experiment setup is created to explore the effect of the location of the mutations in the classification result. The exonic and intronic mutations are used separately. The experiment results are presented in Table 8. When only the exonic mutations are used, the classification performance decreases dramatically to 54.56*%* accuracy and 55.52*%* f-score. This decrease can be dependent on the fact that only 15% of all mutations are exonic. When only intronic mutations are used, the classification performance decreases to 74.39*%* accuracy and 75.54*%* f-score. This relatively lower decrease in the performance can be explained by the vast majority of mutations being intronic. The paired t-test produces a *p*-value < 4.8*e*−02 for the f-score results of the Feed-Forward NN with BM25-tf-rf representation with only intronic and all mutations. The utilization of all exonic and intronic mutations for input representation leads to statistically significant improvement in f-score performance. Similar to recent studies stating that malignancy-driving mutations can also occur outside the coding region [37, 38], our location based comparison results support the need for further research in non-coding variants.

**Table 8:** Machine learning experiment test results on the separated exonic and intronic mutations

Mutation Set	Accuracy	F-Score	Precision	Recall	Roc-Auc	FPR
exonic	54.56 ± 1.18	55.52 ± 0.96	56.52 ± 0.83	54.56 ± 1.18	0.67 ± 0.01	5.44 ± 0.17
intronic	74.39 ± 1.58	75.54 ± 1.30	76.74 ± 1.10	74.39 ± 1.58	0.83 ± 0.01	2.91 ± 0.33
all	76.44 ± 0.66	76.95 ± 0.68	77.48 ± 0.78	76.44 ± 0.66	0.86 ± 0.02	2.75 ± 0.13

## Discussion

The main goal of genomic studies for diseases is to propose target or causal genes. BM25-tf-rf is found to be superior compared to binary, tf-idf and C-score for the representation of DNA mutations for the task of cancer classification. We further analyze our best model (BM25-tf-rf and Feed Forward NN) for the most effective genes in decision making.

**Fig. 3:**
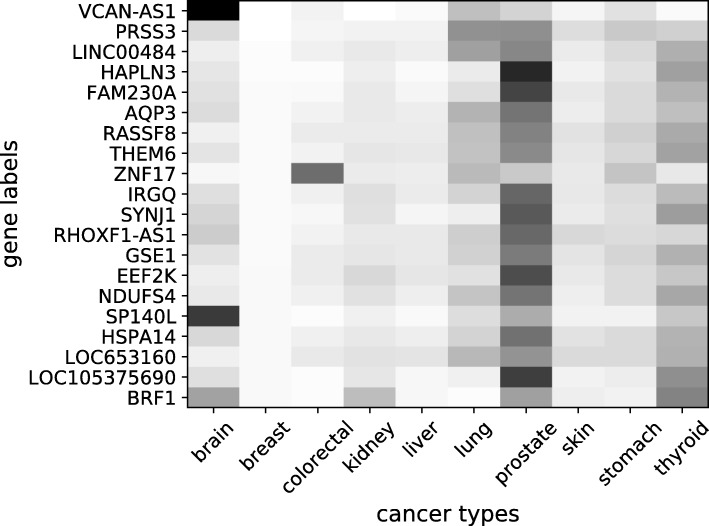
The heat map of the most effective genes in NN with BM25-tf-rf model for breast cancer. A light colored region for a gene and a cancer type can be interpreted as the gene is more effective in the decision of the cancer type. A dark colored region corresponds to less effective state

**Fig. 4:**
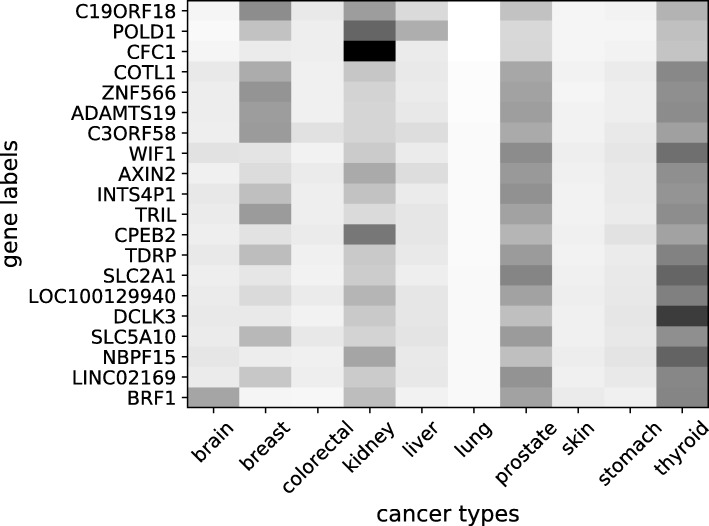
The heat map of the most effective genes in NN with BM25-tf-rf model for lung cancer. A light colored region for a gene and a cancer type can be interpreted as the gene is more effective in the decision of the cancer type. A dark colored region corresponds to less effective state

In Figs. 3 and 4, the heat maps show the most effective genes in the NN model with BM25-tf-rf representation for the classification of the breast and lung cancer types, respectively. The heat maps are constructed by giving one hot vectors, where only one gene feature is set to 1 and the others are set to 0, as input to the 10-fold trained NN classifiers. The output values from the 10 output nodes corresponding to the probabilities for the 10 cancer types, are saved as a heat map for that fold. The final heat map is the average of the folds. Each output of the NN reflects the effect of the labelled gene (set to 1 in the input) to the prediction of the cancer types. If the probability value for a cancer type is high, this means that the labelled gene is more effective in the prediction of that cancer type, since in the NN calculations, the other features are cancelled as their input values are 0. If the probability value for a cancer type is low, this means that the labelled gene doesn’t play an important role in the prediction of that cancer type. The genes that result in high output probability values for a cancer type are taken as more effective and active genes for the prediction of that cancer type.

For the specific heat maps in Figs. 3 and 4, the final heat map is sorted according to the selected class column and 20 genes, which have the highest impact on the prediction of the selected cancer type, are plotted. The values in the heat-maps represent how effective a gene is for the prediction of the corresponding cancer type. The lighter colors represent higher values which refer to more effective state.

In Fig. 3, PRSS3, AQP3, HAPLN3, RASSF8, BRF1 and GSE1 are shown to be effective on classification of breast cancer and there is supporting evidence in the literature for their relatedness to the disease. PRSS3 was found to promote the growth of breast cancer cells [39]. AQP3 is studied in [40]. In [41], HAPLN3 was shown to be among the overexpressed genes for breast cancer. In [42], HAPLN3 was suggested to be involved in the development of breast cancer and to be a biomarker for the treatment of breast cancer. In [43], RASSF8 was proposed to be used in discrimination of benign and malignant breast tumors. In [44], they investigated whether BRF1 expression is increased in the samples of human breast cancer and their results indicated that it is overexpressed in most cases. In [45], it is reported that GSE1 is overexpressed in breast cancer and silencing of GSE1 significantly suppressed breast cancer cells.

The effect of BM25-tf-rf weighting is illustrated through an example for the HAPLN3 and BRF1 genes of two sample patients. Sample-79 is a breast cancer patient and Sample-164 is a brain cancer patient. HAPLN3 and BRF1 have been found relevant to breast cancer in the literature and they are both found to be among the most effective genes for our classification system. But there is no scientific evidence about the relationship of these genes and brain cancer. The BM25-tf-rf value for HAPLN3 in Sample-79 is 1.016021 whereas it is 0.794813 in Sample-164. There is a nearly 28 percent increase in the weight of this gene for the breast cancer sample. For BRF1, the BM25-tf-rf value in Sample-79 is 1.323967 whereas it is 1.085498 in Sample-164. There is a nearly 22 percent increase in the weight of this gene for the breast cancer sample. When we consider the Feed Forward NN classification model, the weights for these gene features is the same for both samples. Therefore, the distinction of predicted cancer type results arise from the difference in BM25-tf-rf weights. This difference effects the decision and helps to distinguish between cancer types.

In Fig. 4, POLD1, COTL1, AXIN2, WIF1 and SLC2A1 (previous symbol GLUT1) are shown to be effective on classification of lung cancer. There are studies in the literature supporting the relatedness of these genes to lung cancer. POLD1 is studied in [46]. In [47] and [48], COTL-1 was proposed to be a biomarker or a therapeutic target for small cell lung cancer (SCLC) patients. In [49], AXIN2 was found to play a major role in modulating lung cancer risk. It was shown that WIF1 had the potential as a methylation biomarker in the diagnosis of non small cell lung cancer (NSCLC) [50]. In [51], it is reported that lung squamous cell carcinoma, a major subtype of NSCLC, exhibits remarkably elevated glucose transporter GLUT1 expression.

Since there is literature evidence for the disease-relatedness of a subset of the most effective genes in classification using NN model with BM25-tf-rf, the other most effective genes that are not studied yet might also have causal roles in cancer development. According to these evidences, NN trained with the BM25-tf-rf representation of the mutations in the VCF files, can also be used for the purpose of finding new candidate genes for cancer types.

## Conclusion

Complex genomic diseases are caused by changes in DNA that alter cell behavior. The impact level of each mutation may be different for various diseases. In order to model this diversity, being inspired from the document representation techniques in the information retrieval domain, we proposed different mutation based statistical genomic data representation schemes.

We utilized VCF files, which contain mutation information in the DNA, for the classification of cancer types as a case study. Cancer, in general, results from a combination of several genomic alterations, which can be addressed in variant calls data. We evaluated the performance of the proposed data representation schemes with a wide range of machine learning algorithms. Our experiment results showed that BM25-tf-rf based representation is more successful at modeling VCF data compared to the binary, tf-idf and C-score based representation schemes. Each cancer type may develop as a result of different gene mutations. The supervised weighting approach of tf-rf successfully reflects this class-mutation relationship. The normalization effect of BM25-tf further improves the classification performance of tf-rf. We investigated the most effective mutated genes in our proposed system for breast and lung cancers. A subset of the resulting genes have also been suggested as causal or target genes in previously published studies, which demonstrates that the proposed approach can also be used to recommend candidate genes.

The introduced data representation models are evaluated for the task of cancer type classification, which is an important problem in bioinformatics, since the appropriate treatment is determined according to the primary site. However, they can also be utilized for other genetic diseases, which we plan to investigate in our future studies.

## Abbreviations

BM25-tf: BM25 definition of term frequency
CADD: Combined annotation dependent depletion
idf: inverse document frequency
KNN: K-nearest neighbor
LR: Logistic regression
NB: Naive bayes
NN: feed-forward multilayer neural network
Perceptron: one-layer-perceptron
tf: term frequency
tf-idf: term frequency-inverse document frequency
tf-rf: term frequency-relevance frequency
rf: relevance frequency
SVM: Support vector machine
TCGA: The cancer genome atlas
VCF: Variant call format
